# Extended endoscopic endonasal approach for resection of a craniopharyngioma in a patient with sphenoid sinus fibrous dysplasia

**DOI:** 10.3171/2026.7.FOCVID26144

**Published:** 2026-10-01

**Authors:** Rodolfo Villalobos-Diaz, Tania M. Jiménez-Molina, Lilian Zavala-Romero, Jorge A. Rochin-Mozqueda, Juan Luis Gómez-Amador, Alfredo Vega-Alarcón, Roberto D. Paz-Lara, Gerardo Y. Guinto-Nishimura

**Affiliations:** 1Department of Neurosurgery, National Institute of Neurology and Neurosurgery Manuel Velasco Suárez, Mexico City; and; 2 Department of Neuro-otology, National Institute of Neurology and Neurosurgery Manuel Velasco Suárez, Mexico City, Mexico

**Keywords:** craniopharyngioma, sphenoid sinus fibrous dysplasia, endoscopic endonasal approach, skull base surgery, optic nerve decompression

## Abstract

This video demonstrates a stepwise endoscopic endonasal strategy for the management of a cystic suprasellar craniopharyngioma coexisting with extensive sphenoid fibrous dysplasia in a 55-year-old man presenting with visual deterioration and neuroendocrine dysfunction. Severe distortion of the sphenoid anatomy required progressive neuronavigation-guided drilling and reconstruction of critical skull base landmarks to safely reestablish the surgical corridor. This strategy enabled successful tumor resection and bilateral optic nerve decompression while preserving the advantages of the endoscopic endonasal route. The technique may be applicable to other complex sphenoid anatomies, including conchal sphenoid sinus variants, when this approach represents the optimal corridor for resection.

The video can be found here: https://stream.cadmore.media/r10.3171/2026.7.FOCVID26144

## Transcript

### 0:24 Clinical Presentation.

We present the case of a 55-year-old man with no significant past medical history, who presented with progressive headache, fatigue, dizziness, cognitive disturbances, and symptoms suggestive of vasopressin deficiency.

### 0:38 Neurological Examination.

On neurological examination, the patient exhibited decreased visual acuity, measuring 20/40 in the right eye and 20/400 in the left eye. Visual field assessment demonstrated a right temporal scotoma, with preservation of the left visual field. No additional neurological deficits were identified.

### 0:59 Neuroimaging Finding.

Contrast-enhanced MRI demonstrated a retroinfundibular suprasellar lesion with solid and cystic components, consistent with a craniopharyngioma. In addition, CT revealed an expansile sphenoid lesion with a characteristic ground-glass appearance consistent with fibrous dysplasia, resulting in bilateral optic canal narrowing.

### 1:21 Surgical Indications for Sphenoid Fibrous Dysplasia.

The management of sphenoid fibrous dysplasia remains controversial. In asymptomatic patients, clinical and radiological follow-up is recommended. Prophylactic optic nerve decompression remains controversial, even in the presence of radiographic optic canal encasement.^1,2^ In contrast, surgical treatment is generally recommended for symptomatic patients, particularly when visual function is compromised.^2–4^

### 1:46 Surgical Techniques.

Several techniques have been described for optic nerve decompression in sphenoid fibrous dysplasia. The endoscopic endonasal approach allows direct optic nerve decompression and may improve visual outcomes, particularly when performed early after symptom onset.^1^

### 2:02 Surgical Indications for Craniopharyngiomas.

Surgical indications for craniopharyngiomas include progressive visual impairment, endocrinological dysfunction, headache or increased intracranial pressure, hydrocephalus, and cognitive or hypothalamic disorders.^1^

### 2:15 Alternative Approaches for Craniopharyngioma Resection.

Multiple surgical approaches for retroinfundibular and third ventricle craniopharyngiomas have been described in the literature.^1,5^ These include pterional approach through lamina terminalis, transfrontal interhemispheric approach through lamina terminalis, interhemispheric transcallosal transforaminal approach, and transventricular or neuroendoscopic approaches. In selected midline lesions, the endoscopic endonasal route offers direct access to the lesion in the suprasellar region while avoiding brain retraction.^5^

### 2:48 Rationale for Simultaneous Surgical Treatment.

Although the craniopharyngioma clearly explained the patient’s vasopressin deficiency through pituitary stalk involvement, the visual impairment was not entirely attributable to the tumor alone. The presence of extensive sphenoid fibrous dysplasia with bilateral optic canal narrowing suggested an additional compressive component contributing to the optic neuropathy. Therefore, simultaneous treatment was considered the most appropriate strategy to address both pathologies responsible for the patient’s symptoms.

### 3:20 Rationale for Endoscopic Endonasal Approach.

Because of the tumor’s retroinfundibular midline location, the endoscopic endonasal approach provided the most favorable corridor for resection while also allowing bilateral optic nerve decompression through the same surgical route.

### 3:34 Sphenoid Fibrous Dysplasia.

Sphenoid fibrous dysplasia significantly increases the complexity of endoscopic endonasal surgery by eliminating the natural sphenoid corridor, distorting anatomical landmarks, and making surgical orientation more challenging.^3,6^

### 3:49 Sphenoid Fibrous Dysplasia—Surgical Strategies.

To overcome these anatomical challenges, meticulous preoperative planning with high-resolution CT and MRI is essential. Progressive navigation-guided drilling of the dysplastic sphenoid bone reconstructs the surgical corridor and enables safe sequential exposure of known critical landmarks.

### 4:07 Sphenoid Fibrous Dysplasia—Surgical Steps.

The senior author advocates a stepwise strategy to restore anatomical orientation. Following ethmoidectomy and exposure of the medial periorbita and orbital apex, the optic nerves are identified and used as key landmarks to guide drilling toward the tuberculum sellae. The sellar floor is then reconstructed using the tuberculum as the primary reference, followed by lateral drilling to define and preserve the cavernous sinus. Once these critical landmarks have been established, the transtuberculum-transplanum approach can be completed safely.

### 4:42 Nasoseptal Flap.

For this case the procedure began with harvesting a right nasoseptal flap for subsequent skull base reconstruction, followed by a posterior septectomy to facilitate binostril surgery. An extensive posterior ethmoidectomy was then performed.

### 4:58 Sphenoid Drilling.

Given the absence of normal sphenoid sinus pneumatization, progressive drilling of the dysplastic sphenoid rostrum was carried out.

### 5:06 Orbital Apex Exposure.

The ethmoid septations were drilled laterally to the level of the lamina papyracea, which was then removed to expose the medial periorbita and orbital apex, employing neuronavigation to confirm the anatomical landmarks throughout the procedure.

### 5:21 Optic Canalization.

Drilling was then continued posteriorly and medially along the expected course of the optic nerve toward the optic chiasm.

### 5:29 Optic Nerve Identification.

The posterior ethmoidectomy was then completed on the right and the optic nerve was subsequently exposed. Once the optic nerves had been identified bilaterally, drilling was continued medially to recreate the sellar tuberculum, which was subsequently confirmed with neuronavigation.

### 5:47 Bilateral Optic Decompression.

The projected courses of both optic nerves were further drilled for decompression. The surgical corridor was then expanded superiorly and inferiorly, using the optic nerves, the estimated location of the sellar tuberculum, and neuronavigation as constant anatomical references to guide and ensure safe drilling.

### 6:07 Sellar Exposure.

Drilling then proceeded inferiorly until the sellar floor dura was exposed, using the location of the sellar tuberculum as the primary anatomical reference. Lastly, drilling was extended superiorly toward the estimated location of the planum sphenoidale until the dura of the anterior cranial fossa was exposed.

### 6:28 Dural Opening.

The planum sphenoidale and sellar dura were then sharply opened in a T-shaped fashion, providing access to the suprasellar and sellar compartments.

### 6:37 Arachnoid Dissection.

The arachnoid was progressively dissected using a combination of sharp and blunt techniques until the structures of the subchiasmatic corridor were identified.

### 6:48 Cyst Decompression.

The lesion was then visualized, and the cystic component was drained to achieve internal decompression and facilitate subsequent dissection.

### 6:57 Solid Tumor Debulking.

The solid component of the lesion was then identified and progressively debulked intracapsularly using bimanual microsurgical technique under direct endoscopic visualization. A combination of sharp and blunt dissection was employed throughout the resection until complete removal of the solid component was achieved.

### 7:18 Intracapsular Inspection.

Intracapsular inspection was then performed to confirm complete removal of the solid component.

### 7:24 Capsular Dissection.

Following removal of the solid component, an extracapsular dissection plane was identified along the posterior aspect of the lesion. This plane was progressively developed with meticulous sharp dissection under direct endoscopic visualization. The tumor capsule was then carefully brought into the surgical corridor and sequentially dissected from the surrounding neurovascular structures using grasping forceps, endoscopic scissors, and microdissectors. Circumferential dissection continued until complete capsular removal was achieved, with partial preservation of the pituitary stalk.

### 8:03 Final Inspection.

Finally, the resection cavity was inspected with a 30° endoscope to confirm completeness of the resection. Inferiorly, the basilar artery bifurcation was identified, and superiorly through the opened third ventricle, the thalamus and foramen of Monro could also be visualized.

### 8:19 Reconstruction.

A multilayer reconstruction was subsequently performed employing autologous fascia lata. An intradural inlay graft was first positioned beneath the dural edges, followed by placement of an extradural onlay graft to achieve watertight closure of the skull base defect. The reconstruction was further reinforced with an autologous fat graft placed in the inferior portion of the surgical corridor to provide structural support for the nasoseptal flap. A Foley catheter balloon was then inflated to support the reconstruction, and the procedure was concluded.

### 8:56 Postoperative Outcome.

The postoperative course was uneventful. Vasopressin deficiency remained well controlled with desmopressin, no CSF leakage was identified after nasal splint removal on postoperative day 5, and the patient was discharged on postoperative day 6. At 3 months’ follow-up, visual improvement was observed, along with significant overall symptomatic improvement.

### 9:19 Imaging Outcome.

Postoperative imaging demonstrated gross-total resection of the craniopharyngioma and satisfactory bilateral optic nerve decompression.

### 9:28 Histopathological Outcome.

Histopathological examination confirmed an adamantinomatous craniopharyngioma and extensive intraosseous fibrosis, consistent with fibrous dysplasia.

### 9:37 Conclusions.

In conclusion, severe sphenoid anatomical distortion should not be considered an absolute limitation to endoscopic endonasal surgery. Progressive drilling and reconstruction of critical skull base landmarks can safely restore the surgical corridor and may be applicable to other challenging sphenoid variants, including conchal sphenoid sinus.^7^ This strategy preserves the advantages of the endoscopic endonasal route when it represents the optimal corridor for craniopharyngioma resection. This case highlights the utility of the endoscopic endonasal approach for the treatment of craniopharyngiomas and bilateral optic nerve decompression.

## Acknowledgments

ChatGPT (OpenAI, GPT-5.5) was used in a limited capacity to corroborate and refine the grammar of text previously drafted by the authors. The scientific content, surgical interpretation, references, and final wording were developed, reviewed, and approved by the authors.

## Disclosures

The authors report no conflict of interest concerning the materials or methods used in this study or the findings specified in this publication.

## Author Contributions

Primary surgeon: Guinto-Nishimura. Assistant surgeon: Jiménez-Molina, Zavala-Romero, Vega-Alarcón. Editing and drafting the video and abstract: Villalobos-Diaz, Jiménez-Molina, Zavala-Romero, Rochin-Mozqueda, Paz-Lara. Critically revising the work: Guinto-Nishimura, Villalobos-Diaz, Zavala-Romero, Gómez-Amador. Reviewed submitted version of the work: Guinto-Nishimura, Villalobos-Diaz, Zavala-Romero. Approved the final version of the work on behalf of all authors: Guinto-Nishimura. Supervision: Guinto-Nishimura, Villalobos-Diaz, Rochin-Mozqueda, Gómez-Amador, Vega-Alarcón.

## Supplemental Information

### Patient Informed Consent

The necessary patient informed consent was obtained in this study.
